# A potential adjuvant chemotherapeutics, 18β-glycyrrhetinic acid, inhibits renal tubular epithelial cells apoptosis via enhancing BMP-7 epigenetically through targeting HDAC2

**DOI:** 10.1038/srep25396

**Published:** 2016-05-05

**Authors:** Taotao Ma, Cheng Huang, Xiaoming Meng, Xiaofeng Li, Yilong Zhang, Shuai Ji, Jun Li, Min Ye, Hong Liang

**Affiliations:** 1 State Key Laboratory of Natural and Biomimetic Drugs, School of Pharmaceutical Sciences, Peking University, 38 Xueyuan Road, Beijing, 100191, China; 2 School of pharmacy, Anhui Medical University, 81 Meishan Road, Hefei, 230032, Anhui, China

## Abstract

Cisplatin, a highly effective and widely used chemotherapeutic agent, has a major limitation for its nephrotoxicity. We recently identified a novel strategy for attenuating its nephrotoxicity in chemotherapy by an effective adjuvant via epigenetic modification through targeting HDAC2. Molecular docking and SPR assay firstly reported that 18βGA, major metabolite of GA, could directly bind to HDAC2 and inhibit the activity of HDAC2. The effects and mechanisms of GA and 18βGA were assessed in CP-induced AKI in C57BL/6 mice, and in CP-treated HK-2 and mTEC cells lines. TUNEL and FCM results confirmed that GA and 18βGA could inhibit apoptosis of renal tubular epithelial cells induced by CP *in vivo* and *in vitro*. Western blot and immunofluorescence results demonstrated that the expression of BMP-7 was clearly induced by 18βGA in AKI models while siRNA BMP-7 could reduce the inhibitory effect of 18βGA on apoptosis. Results of current study indicated that 18βGA inhibited apoptosis of renal tubular epithelial cells via enhancing the level of BMP-7 epigenetically through targeting HDAC2, therefore protecting against CP-induced AKI. These available evidence, which led to an improved understanding of molecular recognition, suggested that 18βGA could serve as a potential clinical adjuvant in chemotherapy.

Cisplatin(CP), which directly interferences with DNA synthesis and the eventual occurrence of apoptosis, is usually used to treat numerous types of cancers. However, nephrotoxicity, a major side effect of chemotherapy, limits its use^1^. Therefore, it is necessary to find effective adjuvant drug to attenuate nephrotoxicity induced by CP. The reversible and dynamic process of epigenetic modification could contribute to a mount of diseases including inflammation and cancer^2^,^3^. Histone deacetylases (HDACs) enzymes balance the acetylation activities of histone acetyltransferases on chromatin remodeling and play essential roles in regulating gene transcription. In the kidney, emerging evidence had demonstrated HDACs contribute to renal damage^4^,^5^. Although Histone acetylation/deacetylation of Acute kidney injury (AKI) is a nascent field, the available information has already provided compelling evidence that chromatin biology plays a critical role in this disease. Thus we recently identified a novel strategy for attenuating nephrotoxicity in chemotherapy via epigenetic modification by targeting HDAC. However, most potent epigenetic drugs show substantial limitations, such as epigenetic nonspecificity (pleiotropic effects) and drug resistance. HDAC inhibitors, such as trichostatin A (TSA) and Vorinostat (SAHA), are subject to epigenetic modification via sulfation, which lead to the buildup of toxic sulfate metabolites of the hydroxy group^6^,^7^,^8^,^9^. For this reason, it is urgent to seek for effective and low-toxic HDAC-targeted drugs.

Licorice is the most frequently used traditional Chinese herbal medicine for thousands of years. Glycyrrhizic acid (GA) is the major active component of Licorice, which possesses a wide range of pharmacological effects such as anti-inflammation and anti-tumor^10^,^11^,^12^ (Supplementary Fig. S1). Increasing evidence showed that GA was found to be very effective in inhibiting 6-hydroxydopamine-induced cytotoxicity in PC12 cells and CCl_4_-induced hepatocyte apoptosis^13^,^14^. Another study by Wu showed GA and 18β-glycyrrhetinic acid (18βGA) played essential roles in CP-induced nephrotoxicity^15^. It is now well accepted that apoptosis of renal epithelial cell is regarded as the pathognomonic lesion of CP-induced AKI. Thus we hypothesized the renoprotective effects of GA might be contributed by the inhibition of renal tubular cells apoptosis.

In this paper, Molecular docking and Surface Plasmon resonance (SPR) assay firstly reported that 18βGA, GA major metabolite *in vivo*, could directly bind to HDAC2 and inhibit the activity of HDAC2. The mechanistic studies of GA and 18βGA were performed in CP-induced AKI in C57BL/6 mice, and in various CP-treated cell lines such as HK-2 and mTEC cells lines. Terminal deoxynucleotidyl transferase dUTP nick end labeling (TUNEL) and flow cytometry (FCM) results confirmed that GA and 18βGA could inhibit apoptosis of renal tubular epithelial cells induced by CP *in vivo* and *in vitro*. Furthermore, Western blot and immunofluorescence results demonstrated that the expression of bone morphogenetic protein-7(BMP-7), a protective molecule in renal inflammation, was clearly up-regulated in AKI models with the addition of GA *in vivo* and 18βGA *in vitro* while siRNA BMP-7 could reduce the 18βGA inhibitory effect on apoptosis. Taken together, this study for the first time identified 18βGA, exerting renoprotective effects related to inhibiting renal tubular epithelial cells apoptosis by restoring BMP-7 signaling through epigenetic regulation in HDAC2-dependant mechanism. These available evidence, which led to an improved understanding of molecular recognition, suggested that 18βGA could serve as a potential clinical adjuvant in chemotherapy.

## Results

### 18βGA directly binds to HDAC2 by Molecular docking and SPR assay

**Figure 1:**
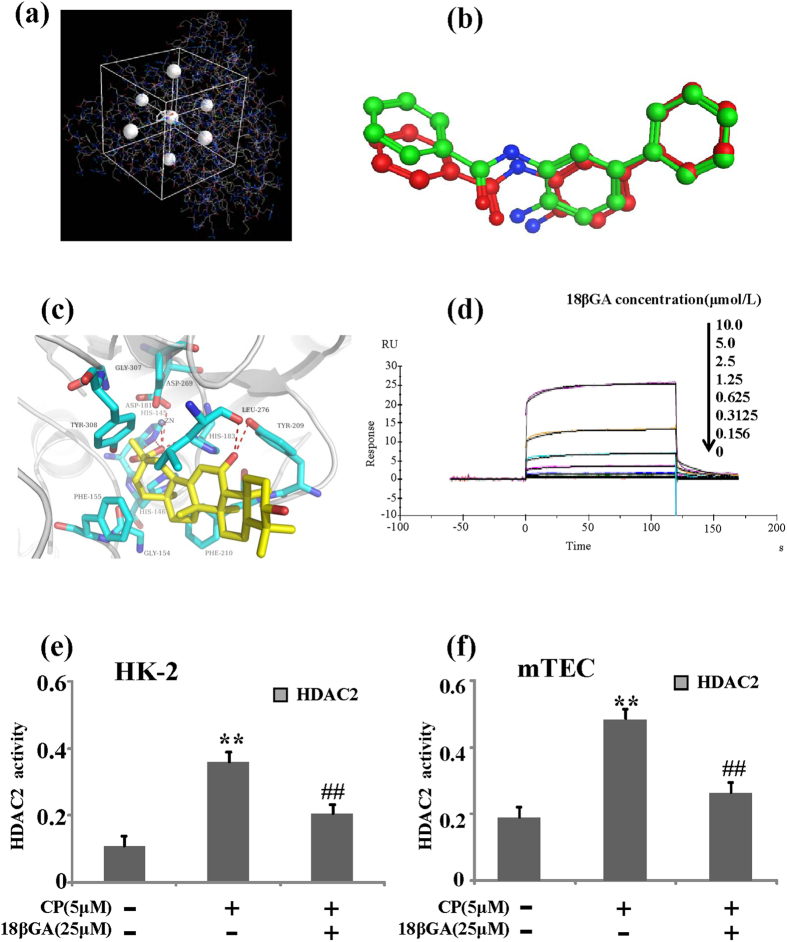
18βGA directly binds to HDAC2. (**a**) The center of native ligand (LLX, N-(4-aminobiphenyl-3-yl)benzamide,) defined the binding pocket. (**b**) Cognate ligand within the binding pocket of HDAC2 by MOE-Dock. (**c**) 18βGA was located in catalytic center of HDAC2 (Zn^2+^, HIS145, HIS146, and HIS183). (**d**) The binding affinity of 18βGA with HDAC2 was measured by SPR technology. (**e**) Effects of 18βGA on the activity of HDAC2 induced by CP in HK-2 cells. (**f**) Effects of 18βGA on the activity of HDAC2 induced by CP in mTEC cells. Data are represented as mean ± SD of three independent experiments. *p < 0.05, **p < 0.01 vs. control group, ^#^p < 0.05, ^##^p < 0.01 vs. CP alone.

In docking study, re-docking protocol was performed on co-crystallized structure of HDAC2 (PDB entry: 3MAX). The competency assessment of each re-docked pose was evaluated by considering the Root-mean-square deviation (RMSD) values. Most of RMSD values between docking poses of native ligand and experimental pose are less than 2.0 Å. All of these results suggested that MOE-Dock was able to produce the most convincing re-docking results for cognate ligand within the binding pocket of HDAC2 (Fig. 1b). As shown in Fig. 1c, 18βGA was located in catalytic center of HDAC2 (Zn^2+^, HIS145, HIS146, and HIS183). The carboxy group of 18βGA was inserted into catalytic center of HDAC2 to form five hydrogen bonds with the side chain of HIS145, HIS146, and HIS183 and Zn^2+^.Another two hydrogen bonds were formed between hydroxyl of 18βGA and the side chain of LEU276 and TYR209. Moreover, the hydrophobic of 18βGA was surrounded by the hydrophobic residues (PHE155, TYR308, GLY154, PHE210, LEU276, and TYR209, Fig. 1c), suggesting hydrophobic interactions would be formed between 18βGA and HDAC2. All of these molecular recognition results were consistent with our bioassay results.

To further confirm the interaction of 18βGA with HDAC2, the SPR-based Biacore T200 biosensor was used to measure the binding affinity of 18βGA with HDAC2. The HDAC2 protein was immobilized on sensor chip, and binding responses in RUs were continuously recorded and presented graphically as a function of time in sensorgrams. The association of compound with HDAC2 was evaluated using the equilibrium dissociation constant (KD) by fitting the sensorgram with a 1:1 (Langmuir) binding fit model. As shown in Fig. 1d and Supplementary Table S1, 18 βGA had a high binding affinity towards HDAC2 in a concentration-dependent manner. The dissociation equilibrium constant (KD) was calculated to be 0.6131 μM.

Recent findings of over-expression and/or increased activity of histone deacetylases (HDACs) in cancer cells and low basal level in normal cells made HDACs potential therapeutic targets for cancer treatment^16^. In order to inspect 18βGA influence on HDAC activity, deacetylase activity was measured by a commercial colorimetric HDAC2 assay kit. It was interesting to explore that the activity of HDAC2 was significantly blunted by 18βGA in CP-treated HK-2 and mTEC cells (Fig. 1e,f).

### Effects of GA on mice with CP-induced AKI

**Figure 2:**
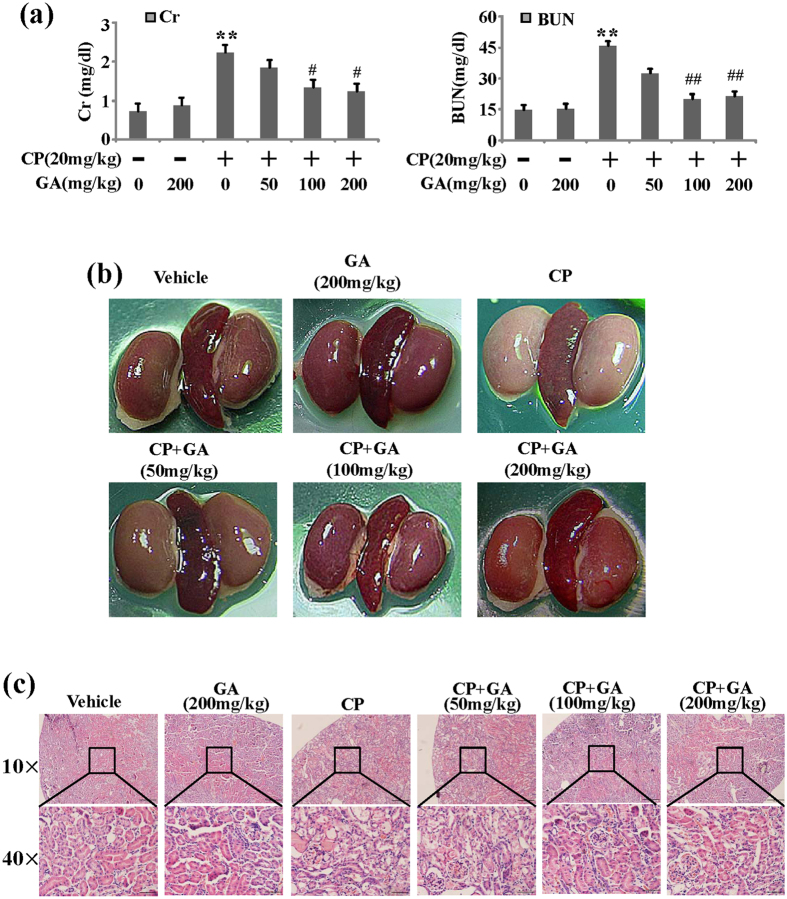
Effects of GA on acute kidney injury after CP administration. (**a**) Effects of GA on serum Cr and BUN. Data are represented as mean ± SD of 10 animals of each group. *p < 0.05, **p < 0.01 vs vehicle group; ^#^p < 0.05, ^##^p < 0.01 vs CP-induced group. (**b**) Representative macroscopic appearances of the kidneys. (**c**) Representative histological changes in kidneys obtained from mice of different groups. The sections shown were harvested 12 h after CP injection and stained with H&E. Magnification: ×10 and ×40.

To investigate the effects of GA on AKI induced by CP, we treated C57BL/6 mice with 0.5% CP intraperitoneal injection. As shown in Fig. 2a, both BUN and Cr levels were significantly increased in CP induced group compared with vehicle group. The increases were significantly attenuated by treatment with GA while GA treatment alone had no significant effects on BUN and Cr levels. These results indicated that GA had a protective effect on CP-induced AKI without nephrotoxicity at a high dose of 200 mg/kg.

Histopathological change was a direct indication of renal injury. This study used both morphological changes in the appearance of the kidneys and renal tubular lesions as indicators to evaluate renal injury. The macroscopic appearances showed CP induced significant lesions that produced whitening of the kidneys in mice while the GA-treated sample groups exhibited a hyperemic dark-maroon color (Fig. 2b).

The H&E-stained renal tissues appeared to have normal kidney tubules in the vehicle group samples. In contrast, it was demonstrated that severe renal tubular injury was caused by CP, such as the edema of renal tubular epithelial cells, dilation of renal capsule cavity, the epithelial cells of the local focal necrosis collapse. However, pretreatment with GA (50, 100, 200 mg/kg) could diminish the state of injury kidney, such as epithelial atrophy and necrosis. The observation was further confirmed at the score of kidney pathological damage (Fig. 2c).

**Figure 3:**
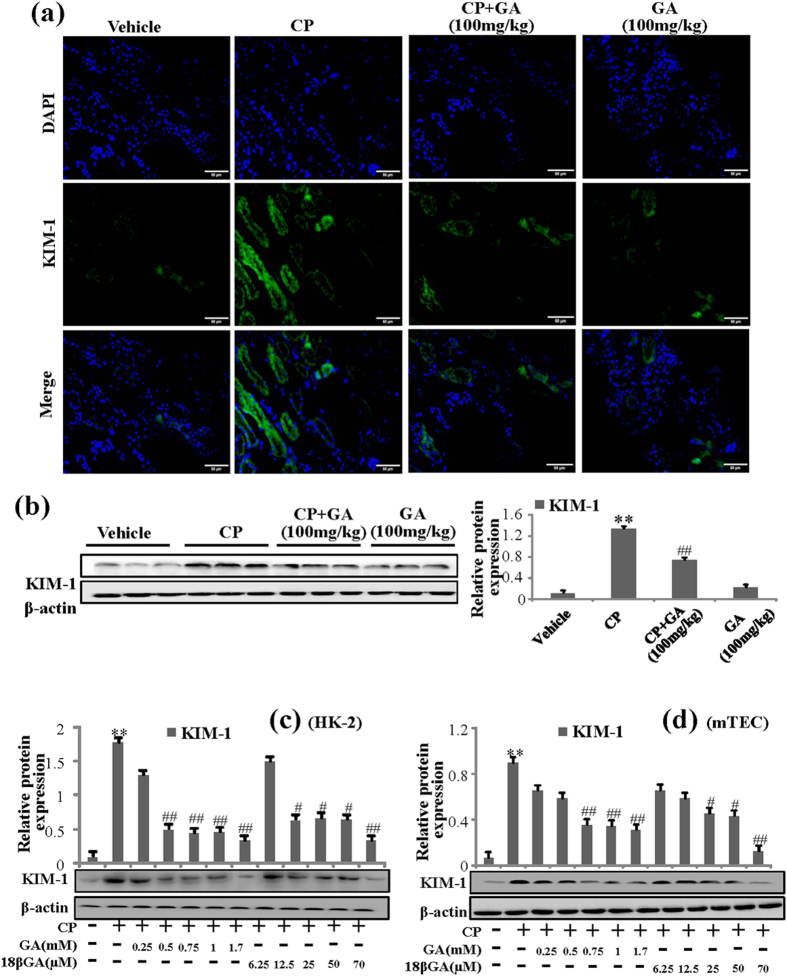
Effects of GA and 18βGA on expression of KIM-1 *in vivo* and *in vitro*. (**a**) Immunofluorescence analysis of KIM-1 protein treated with GA. Results were obtained under medium antibody concentration, the results were similar from three independent experiments. (**b**) Effects of GA on the expression of KIM-1 in the CP-induced AKI. Protein extracts were obtained from kidney tissues and levels were detected by Western blot analysis. Data represent the means ± SD from three independent experiments. *p < 0.05 vs vehicle group, ^#^p < 0.05 vs CP-treat group. (**c**) Effects of different concentrations GA and 18βGA on expressions of KIM-1 induced by CP in HK-2 cells. (**d**) Effects of different concentrations GA and 18βGA on expressions of KIM-1 induced by CP in mTEC cells. Data are represented as mean ± SD of three independent experiments. *p < 0.05, **p < 0.01 vs. control group, ^#^p < 0.05, ^##^p < 0.01 vs. CP alone.

Kidney injury molecule-1(KIM-1) is a biomarker of kidney injury^17^. Immunofluorescence staining analysis revealed that KIM-1 protein expression was clearly upregulated in CP-treated group compared to the vehicle kidney while GA could decrease the expression of KIM-1 protein in Fig. 3a. Similar findings were also demonstrated by Western blot analysis (Fig. 3b).

GA could be transformed into 18β-glycyrrhetinic acid (18βGA) via biotransformation^18^. At first, compounds GA and 18βGA were evaluated for their antiproliferative activities against mTEC and HK2 cell lines. The cells were allowed to proliferate in the presence of tested material for 48 h, and the results were reported in terms of IC50 values (Supplementary Table S2). It was obvious from Supplementary Table S2 that compound 18βGA exhibited higher activities against mTEC and HK2 cell lines with IC50 value of 0.089 and 0.073 mM respectively, surpassing that of GA. Thus we chose GA 0.05, 0.1, 0.2, 0.4, 0.8 mM and 18βGA 2.5, 5, 10, 20, 30 μM to further evaluate the roles on KIM-1. It was demonstrated that both GA and18βGA could decrease CP-induced high levels of KIM-1 in the cell lines of HK-2 (Fig. 3c) and mTEC (Fig. 3d). Interestingly, compared to GA, 18βGA showed stronger pharmacological nephroprotection activities. Thus, 18βGA was chosen to discuss the mechanisms in AKI induced by CP.

### GA and 18βGA inhibit CP-induced apoptosis of renal tubular epithelial cells *in vivo* and *in vitro*

**Figure 4:**
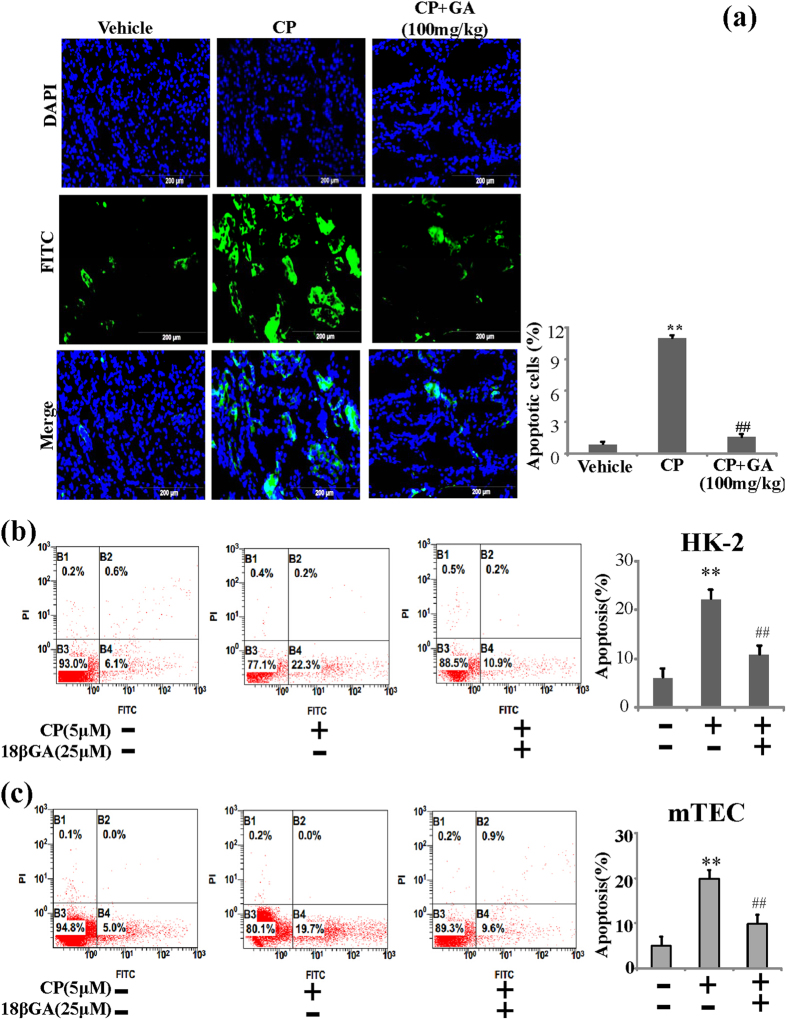
Effects of GA and 18βGA on apoptosis of renal tubular epithelial cells *in vivo* and *in vitro*. (**a**) Representative images of TUNEL staining in different group. Scale bars show 200 μm. (**b**) Apoptosis of HK-2 were analyzed by flow cytometry with Annexin V-FITC and PI staining. (**c**) Apoptosis of HK-2 were analyzed by flow cytometry with Annexin V-FITC and PI staining. A representative image of the three independent experiments were demonstrated. *p < 0.05, **p < 0.01 vs. control group, ^#^p < 0.05, ^##^p < 0.01 vs. CP alone.

Renal tubular epithelial cells apoptosis is the common histopathological feature of cisplatin nephrotoxicity^19^. TUNEL stain was used to detect apoptotic cell death in the CP-treated AKI. It was demonstrated that numerous TUNEL positive cells were observed in CP-treated AKI in contrast with vehicle kidney, while addition of GA could significantly decrease TUNEL positive cells. Similar observation was further confirmed at the score of kidney apoptosis (Fig. 4a).

To further investigate the anti-apoptotic effect of GA, we performed studies on 18βGA, major metabolite of GA, in HK-2 and mTEC cell lines. At first, apoptosis assay by flow cytometric analysis was carried out, and the results indicated that 18βGA showed high activity against apoptosis treated by CP (Fig. 4b,c).

**Figure 5:**
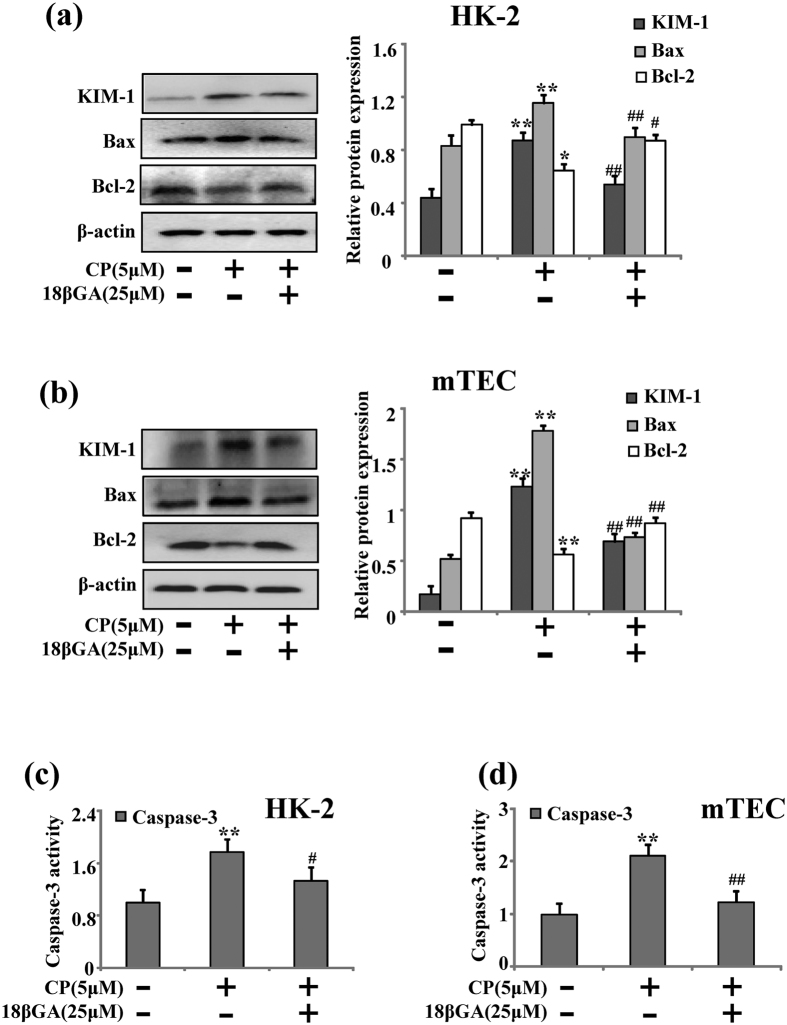
Effects of 18βGA on Bcl-2, Bax and caspase-3. (**a**) Effects of 18βGA on expression of KIM-1, Bcl-2 and Bax induced by CP in HK-2 cells. (**b**) Effects of 18βGA on expression of KIM-1, Bcl-2 and Bax induced by CP in mTEC cells. (**c**) Effects of 18βGA on the activity of caspase-3 induced by CP in mTEC cells. (**d**) Effect of 18βGA on the activity of caspase-3 induced by CP in mTEC cells. Data are represented as mean ± SD of three independent experiments. *p < 0.05, **p < 0.01 vs. control group, ^#^p < 0.05, ^##^p < 0.01 vs. CP alone.

B-cell lymphoma-2 (Bcl-2) family, regulators of programmed cell death, play a central role in renal tubular epithelial cells apoptosis during cisplatin nephrotoxicity^20^. Thus the protein levels of Bcl-2 and Bax were detected by Western blot analysis. It was demonstrated that 18βGA could decrease the high expressed protein of Bax and increased Bcl-2 protein expression induced by CP in HK-2 and mTEC cells (Fig. 5a,b). Interestingly, the activity of caspase-3 inhibited by 18βGA was also detected (Fig. 5c,d).

### GA and 18βGA suppress BMP-7 expression in mice with AKI *in vivo* and *in vitro*

**Figure 6:**
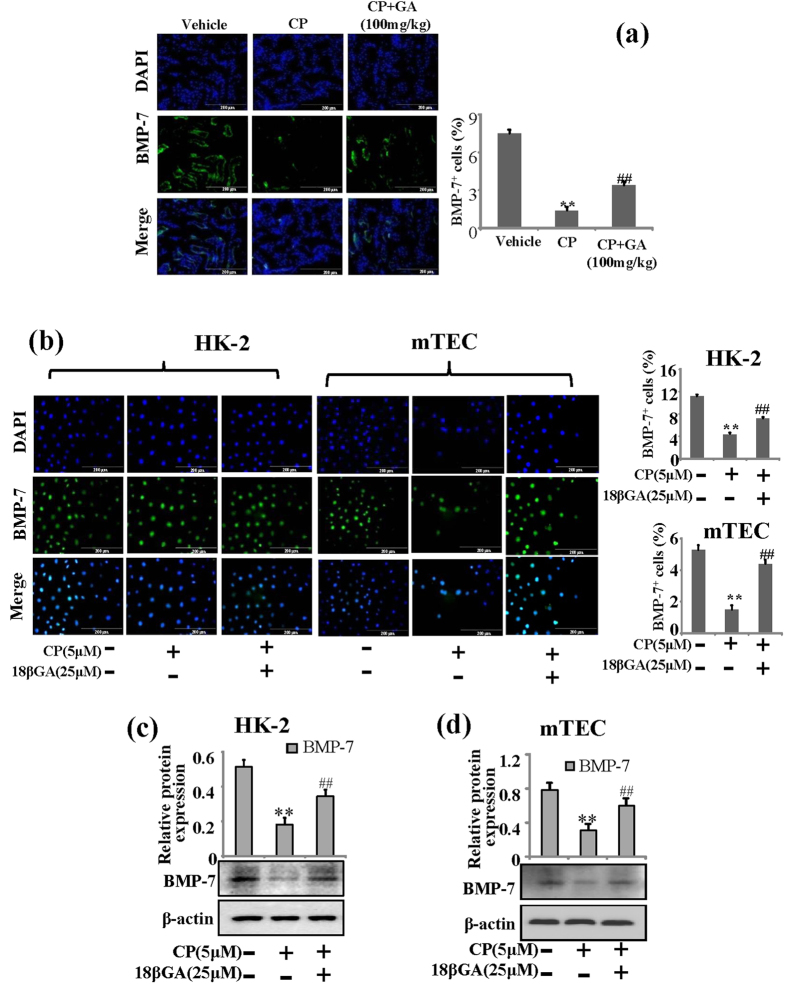
Effects of GA and 18βGA on expressions of BMP-7 *in vivo* and *in vitro*. (**a**) Immunofluorescence analysis of BMP-7 protein treated with GA *in vivo*. Results were obtained under medium antibody concentration. **p < 0.01 vs. control group, ^##^p < 0.01 vs. CP alone. (**b**) Immunofluorescence analysis of BMP-7 protein treated with 18βGA in HK-2 cells and mTEC cells. (**c**) Western blot analysis of 18βGA on expression of BMP-7 induced by CP in HK-2 cells and mTEC cells. Data are represented as mean ± SD of three independent experiments. *p < 0.05, **p < 0.01 vs. control group, ^#^p < 0.05, ^##^p < 0.01 vs. CP alone.

Over the last decade BMP-7 has emerged as a critical renal protective protein that safeguards the kidney against a variety of stimuli that caused renal injury^21^. As shown in Fig. 6a, immunofluorescence staining demonstrated that the expression of BMP-7 was decreased in CP induced AKI while GA could increase its expression in GA treatment group (100 mg/kg). These observations were further confirmed in HK-2 and mTEC cells by Western blot and immunofluorescence staining test. It was suggested that the expression of BMP-7 was reduced when compared with addition of 18βGA *in vitro* (Fig. 6b,c).

**Figure 7:**
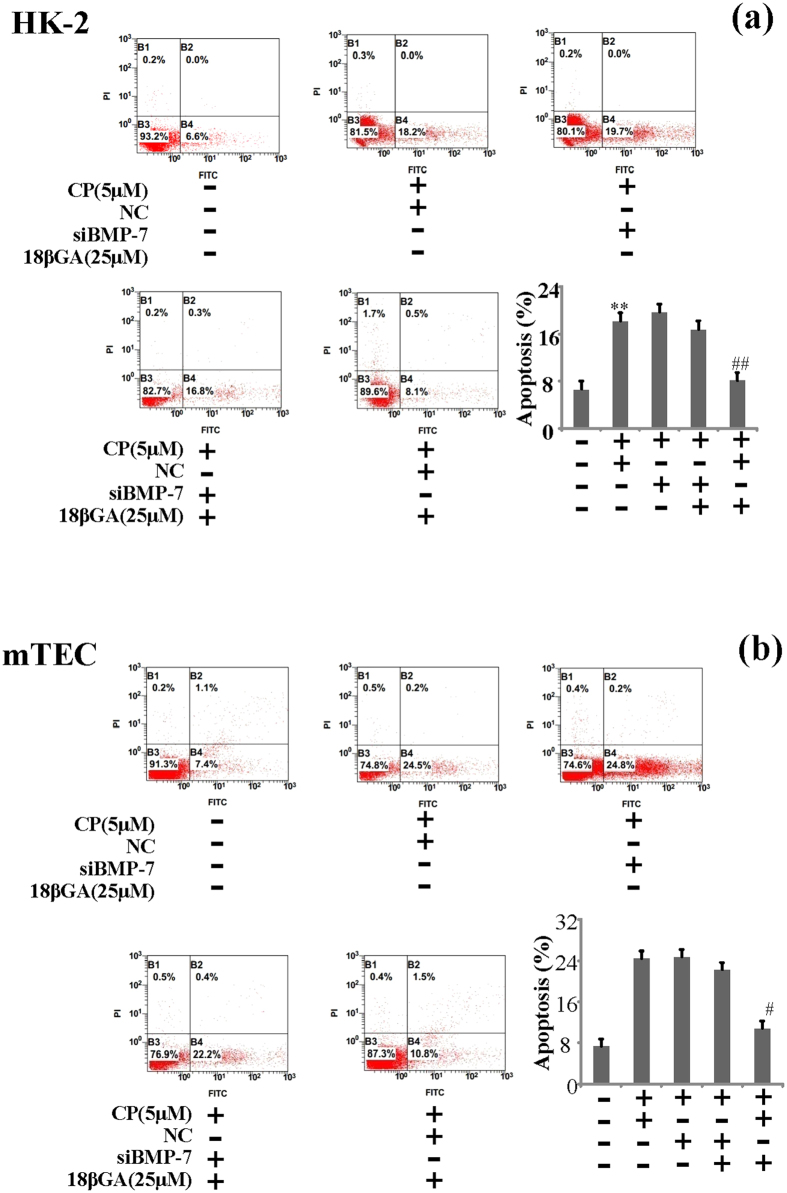
Effects of 18βGA on apoptosis of HK-2 and mTEC cells induced by CP in BMP-7 knockdown cells. (**a**) Apoptosis was analyzed by flow cytometry with Annexin V-FITC and PI staining in BMP-7 knockdown HK-2cells. (**b**) Apoptosis was analyzed by flow cytometry with Annexin V-FITC and PI staining in BMP-7 knockdown mTEC cells. A representative image of the three independent experiments was demonstrated. *p < 0.05, **p < 0.01 vs. control group, ^#^p < 0.05, ^##^p < 0.01 vs. NC.

**Figure 8:**
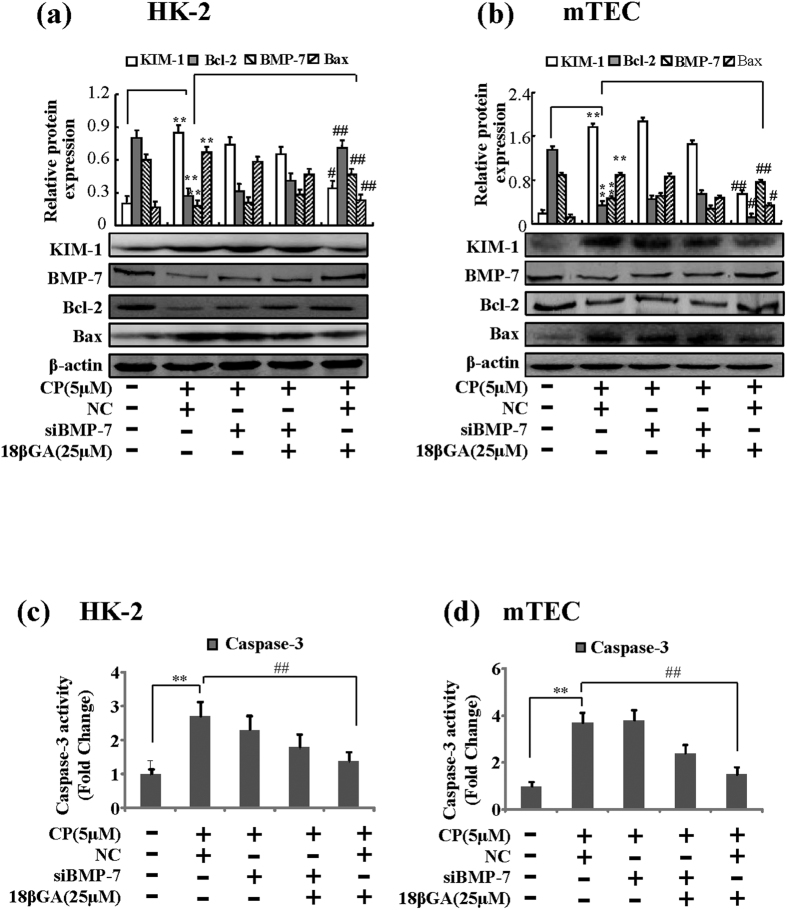
Effects of 18βGA on Bcl-2, Bax and caspase-3 in BMP-7 knockdown cells. (**a**) Effects of 18βGA on expression of KIM-1, Bcl-2 and Bax induced by CP in BMP-7 knockdown HK-2 cells. (**b**) Effects of 18βGA on expression of KIM-1, Bcl-2 and Bax induced by CP in BMP-7 knockdown mTEC cells. (**c**) Effects of 18βGA on the activity of caspase-3 induced by CP in BMP-7 knockdown HK-2 cells. (**d**) Effect of 18βGA on the activity of caspase-3 induced by CP in BMP-7 knockdown mTEC cells. Data are represented as mean ± SD of three independent experiments. *p < 0.05, **p < 0.01 vs. control group, ^#^p < 0.05, ^##^p < 0.01 vs. NC.

To determine whether BMP-7 regulated CP-induced apoptosis in renal tubular epithelial cells, HK-2 and mTEC cells were transfected at high efficiency with siRNAs designed to inhibit BMP-7 expression. Firstly, flow cytometric analysis indicated that the numbers of apoptosis renal tubular epithelial cells transfected with BMP-7 siRNA were largely increased induced by CP, unexpectedly, it couldn’t be inhibited when treating with 18βGA (Fig. 7). Similar findings were shown by Western blot that protein level of Bax and the activity of caspase-3 were increased in transfected with BMP-7 siRNA cells while the level of Bcl-2 protein was decreased (Fig. 8a–d).

Previous study by Marumo *et al.* reported HDAC2 could regulate expression of BMP-7. The histone deacetylase inhibitor trichostatin A (TSA), a high potency and specificity inhibition of HDAC, was used in this study. Western blot results indicated TSA could increase BMP-7 expression in HK-2 and mTEC cells treated by CP and similar findings were found in 18βGA group (Supplementary Fig. S2).

Thus findings *in vitro* supported that 18βGA inhibited apoptosis by enhancing BMP-7 expression via targeting HDAC2 in response to CP.

## Discussion

Emerging evidence had demonstrated great therapeutic contributions to treating cancer since the US Food and Drug Administration (FDA) approved CP as a chemotherapy drug in 1978. It is now clear that chemotherapy drugs usually cause side effects in patients leading to cessation of treatment in current cancer treatment^22^. Nephrotoxicity is the major clinical side effect of CP and increased serum Cr and BUN levels can be detected in patients treated with CP. Therefore, it is necessary to find effective adjuvant drug to attenuate CP-induced nephrotoxicity. Natural products often play an adjuvant role in clinical therapy^23^,^24^. In Traditional Chinese Medicine (TCM) Licorice is commonly used in herbal formulas to harmonize other ingredients. The compendium of Materia Medica (Bencao Gangmu) states that Licorice can act as a beneficial agent in prevention of the progression of disease. GA, a triterpenoid saponin glycoside, is the major active component of Licorice. On the basis of modern pharmacological and clinical studies, it is shown that GA possesses various biological actions, such as antioxidant, hepatoprotective, anti-inflammatory, immunomodulatory activity and so on^25^,^26^. On the basis of our study, it was demonstrated that GA could ameliorate CP-induced AKI as supported by results of Cr, BUN analysis, the appearances of the kidneys and histological observations. Available evidence had suggested Kidney Injury Molecule-1 (KIM-1) was a novel histological biomarker for human renal proximal tubule injury^27^,^28^. Another study by Tekce *et al.* also reported KIM-1 might predict cisplatin-induced AKI in early stages with high sensitivity and specificity^29^. In this study, our results indicated that GA could decrease CP-induced high levels of KIM-1. Previous studies have shown that GA could be transformed into 18βGA via biotransformation. This process could be completed by intestinal bacteria that perform glycolysis. Therefore in this study, it was demonstrated that GA and 18βGA both decreased CP-induced high levels of KIM-1 in the cell lines of HK-2 and mTEC. These observations were further supported by our findings that 18βGA showed stronger pharmacological nephroprotection activities than GA.

It is well accepted that renal tubular epithelial cells are the major sites of cell injury and apoptosis is the common histopathological feature during cisplatin nephrotoxicity. Available evidence suggested that the major apoptotic pathway in cisplatin nephrotoxicity was the intrinsic or mitochondrial pathway. The anti-apoptotic gene, Bcl-2, and the pro-apoptotic gene, Bax, played key roles in cisplatin-induced AKI. Encouragingly, Jiang *et al.* reported that treatment with cisplatin resulted in the decrease of Bcl-2, whereas the levels of Bax were increased^30^. Moreover, knockout of Bax diminished renal tubular epithelial cells apoptosis during cisplatin nephrotoxicity^31^. In the current study, the number of TUNEL-positive cells increased significantly after the cisplatin injection whereas GA could reduce the number of cells positive for TUNEL staining. Furthermore, the expression of pro-apoptotic Bax was increased by cisplatin treatment while 18βGA significantly suppressed the level of Bax. Conversely, the expression of anti-apoptotic Bcl-2 was significantly increased after 18βGA treatment. Thereby 18βGA might exert protective effects on cisplatin-induced renal tubular epithelial cells apoptosis through modulation of the cisplatin-induced expression of Bcl-2 family proteins, which was further supported by the findings that the cisplatin-mediated activity of caspase-3 was also attenuated by 18βGA.

In order to gain more understanding of the structure-activity relationships observed at the 18βGA, molecular modeling studies were carried out. It was demonstrated that molecular docking of 18βGA was performed on the binding model based on the HDAC2 complex structure using the Discovery Studio 3.5 software. Histone deacetylases (HDACs) are a large family of evolutionarily conserved enzymes which balance the acetylation activities of histone acetyltransferases on chromatin remodeling and play essential roles in regulating gene transcription^32^. Available evidence suggested that HDACs were critically involved in kidney diseases^33^,^34^,^35^. Noh *et al.* reported Histone deacetylase-2 was a key regulator of diabetes and transforming growth factor-beta1-induced renal injury^36^. A recent study showed that decreasing HDAC2 and HDAC5 expression levels were in parallel with increasing acetyl histone H3 and associated with the renoprotective effect of dexmedetomidine in sepsis-induced AKI^37^. To further verify the inhibitory effect of 18βGA on the appotosis of HK-2 and mTEC cells *in vitro*, surface plasmon resonance (SPR) technology-based assay was performed to study the binding affinity of 18βGA for HDAC2. SPR biosensors represent the most advanced and developed optical label free biosensor technology. It is a powerful detection and analysis tool that has vast applications in biotechnology, medical diagnostics and drug screening. The results of SPR showed the 18βGA had a highly specific binding affinity towards HDAC2 with the KD = 0.613 μM using Biacore T200. These findings were further supported that cisplatin-mediated the activity of HDAC2 was also attenuated by 18βGA.These observations indicated that the renoprotective mechanisms might be largely contributed by the critical anti-apoptotic roles of 18βGA, which related to targeting HDAC2, leading to an improved understanding of molecular recognition.

A recent study by Manson *et al.* reported that HDAC-dependent repression of BMP-7 transcription was a critical event during the pathogenesis of renal injury in obstructive nephropathy^38^. *In vitro* studies confirmed that histone acetylation was involved in the downregulation of E-cadherin and upregulation of BMP-7^39^. Similarly, Hsing *et al.* also found that dexmedetomidine protected against septic acute kidney injury through inhibiting HDAC2 and HDAC5 and increasing BMP-7^37^. On the basis of these experiments, it was suggested that proximal tubule recovery following renal ischemia might be modulated by the activitation of HDAC, perhaps through the reactivation of BMP7. It is now well accepted that bone morphogenetic protein-7(BMP-7), a key member in the TGF-β superfamily, plays an important role in kidney diseases^21^,^40^. Increasing evidence showed that BMP-7 played a protective role on renal inflammation. Under disease conditions, expression of renal BMP-7 was significantly down-regulated, as detected in diabetic nephropathy and ischaemic acute kidney injury. In addition, administration of rhBMP-7 (recombinant human BMP-7) or overexpression BMP-7 was able to suppress renal fibrosis during diabetic nephrophay. In this study, BMP-7 was downregulated in CP-induced AKI and in cultured HK-2, mTEC cells after CP-treated. It was noted that administration of GA significantly increased BMP-7 expression. Similarly, 18βGA also increased the expression of BMP-7 in CP-treated HK-2 and mTEC cells. Furthermore, knockdown of BMP-7 by RNAi, the antiapoptotic effect of 18βGA could be reduced, suggesting a critical role for BMP-7 in negatively regulating apoptosis in response of CP-induced AKI.

In conclusion, this study provided novel evidence for a protective role of GA in CP-induced AKI in C57BL/6 mice. Interestingly, 18βGA inhibited apoptosis of renal tubular epithelial cells via enhancing level of BMP-7 epigenetically through targeting HDAC2, therefore protecting against CP-induced AKI (Supplementary Fig. S3). This study provided evidence of possible epigenetic effects of GA on renal protection during AKI induced by CP. Results from this study also implicated that targeting HDAC2 might be a specific therapeutic approach for the treatment of progressive acute kidney injury.

## Methods

### Material Reagents

Cisplatin (CP), glycyrrhizic acid ammonium salt (GA), 18β-glycyrrhetinic acid (18βGA), Dimethyl sulfoxide (DMSO), MTT (3-(4, 5-dimethylthiazol-2-yl)-2, 5-diphenyltetrazoliumbromide) and all other chemicals were obtained from Sigma-Aldrich (St. Louis, MO, USA). BMP-7, KIM-1, Bcl-2 and Bax antibodies were purchased from Santa Cruz (CA, USA). β-actin antibody and secondary antibodies for goat anti-rabbit IgG HRP and rabbit anti-goat IgG HRP were purchased from Bioworld Technology (Nanjing, China). Creatinine (Cr) and Blood Urea Nitrogen (BUN) assay kit were purchased from Siemens (NY, USA).

### Molecular docking

The structure of HDAC2 used in the docking calculations was retrieved from the Protein Data Bank (PDB entry: 3MAX)^41^. All water molecules were removed and the chain A of protein was kept and assigned the Amber force field. The initial structure of 18βGA was optimized using the MMFF force field. The Powell method was used for energy minimization via default parameters set in Discovery Studio 3.5.

Vina docking encoded in PyRx software was employed to identify the potential binding of 18βGA to HDAC2. The binding pocket was defined by the center of native ligand (LLX, N-(4-aminobiphenyl-3-yl) benzamide, Fig. 1a). Docking parameters are set to default values. All docked poses of 18βGA were clustered using a tolerance of 2 Å for the root mean square deviation (RMSD) and ranked on the basis of the binding docking energies. The lowest energy conformation in the most populated cluster was chosen for further study.

### Surface plasmon resonance (SPR) technology-based assay

The potential for direct binding of glycyrrhetinic acid to HDAC2 was investigated using a fully automated SPR-based Biacore T200 instrument. During the experiment, HDAC2 was immobilized on a CM5 sensor chip according to the Biacore manual. 18βGA was serially diluted with HBS buffer [10 mmol/L HEPES, 150 mmol/L NaCl, 3 mmol/L EDTA and 0.05% (v/v) surfactant P20] to a final concentration of 0.1% DMSO (v/v). The samples were injected into the channels at a flow rate of 30 μl/min and then washed with HBS buffer. The binding RU (Response Unit) values of 18βGA to HDAC2 were recorded directly by the Biacore T200 instrument and calculated by subtracting the signal from the vehicle (0.1% DMSO).

### Animal treatment

C57BL/6 mice supplied by the Experimental Animal Center of Anhui Medical University were used to establish acute kidney injury model. All the animal experiments were performed in accordance with the Regulations of the Experimental Animal Administration issued by the State Committee of Science and Technology of China. Efforts were made to minimize the number of animals used and their suffering. Animals were maintained in accordance with the Guides of Center for Developmental Biology, Anhui Medical University for the Care and Use of Laboratory. Animals and all experiments used protocols approved by the institutions’ subcommittees on animal care. The mice were randomly divided into five groups (n = 10 per group): vehicle group, model group and GA treatment groups (50, 100, 200 mg/kg). In GA treatment group, 50, 100, 200 mg/kg of GA was given via gavage for three days before the AKI mice model was induced by CP. The mice of model group and GA treatment group were injected intraperitoneally with a single dose of CP (20 mg/kg) on the fourth day, to induce acute kidney injury while vehicle groups received the same volume of vehicle normal saline intraperitoneally. All mice were sacrificed under light ether anesthesia after 24 h of CP treatment and samples of blood and kidneys were collected. Blood was centrifuged at 2000 rpm for 10 min and the separated sera were collected for measurement of renal function tests. Kidneys were decapsulated and washed in cold isotonic saline. The cortex was carefully separated from medulla as described earlier by Banday *et al.*^42^.

### Cell Culture

HK2 and mTEC cells were cultured in DMEM/F-12 (HyClone, USA) with supplemented with 10% (vol/vol) heat-inactivated fetal bovine serum (Mmerck Millipore, Germany) at 37 °C humidified incubator under 5% CO_2_. This study was approved by the Ethics Committee of Anhui Medical University, Hefei, China. The methods were carried out in accordance with the approved guidelines and regulations.

### Cr and BUN assay kits

The concentrations of Cr and BUN in serum from C57BL/6 mice with the acute kidney injury were determined by Cr and BUN assay kits according to the manufacturer’s instructions.

### Histopathology

Renal tissues of mice were fixed in 4% paraformaldehyde for 24 h immediately following sacrifice, processed for histological examination according to a conventional method, and stained with hematoxylin and eosin (H&E).

### MTT assay

Cell viability assay was determined by MTT. HK2 and mTEC were seeded into each well of 96-well culture plates and then cultured in DMEM/F-12 for 24 h. After culture, 20 μl of MTT solution (5 mg/ml) was added to each well, and the plates were then incubated at 37 °C for another 4 h. The optical density (OD) was measured at 492 nm after removing the supernatant and shaking with 150 μl DMSO by Multiskan MK3 (Biotek, USA).

### Immunofluorescence Staining

Renal tissues of mice were fixed in 4% paraformaldehyde for 24 h immediately following sacrifice, staining was performed with rabbit anti-KIM-1, goat anti-BMP-7. Similarly, cultured HK2 and mTEC cells were induced by CP(5 μM) and 18βGA were used in experiments, then cells were fixed with acetone. Staining was performed with goat anti-BMP-7. Counterstaining of secondary antibody and nuclei was performed with 4′, 6-diamidino-2-phenylindole (DAPI; Biyuntian biological technology, Shanghai, China).

### Western Blot

Whole extracts were separated by 10 or 12% sodium dodecyl sulfate polyacrylamide gel electrophoresis (SDS-PAGE), transferred to a polyvinylidene difluoride (PVDF) membrane, which were incubated with primary anti-bodies against BMP-7 and KIM-1 (1:500, Santa Cruz, CA, USA), Bcl-2 and Bax (1:200, Bioss, China). The membranes were then washed in TBS/Tween 20 and incubated with secondary antibodies correspondingly. After extensive washing in TBS/Tween 20, protein bands were visualized with ECL-chemiluminescent kit (ECL-plus, Thermo Scientific).

### Assay of caspase-3 activity

The activity of caspase-3 was measured by using the caspase-3 activity kit (Bestbio, China), according to the manufacturer’s instruction. Assays were performed on 96-well microtiter plates. Ten microliter protein extracts, 90 μl of reaction buffer, and 10 μl of caspase substrate were added by turns. Then, the protein extracts were incubated at 37 °C for 2–3 h. Samples were measured with Multiskan MK3 (Biotek, USA) at an absorbance of 405 nm.

### TUNEL assay

Tumor apoptosis was measured using a TUNEL assay (Keygen Biothech, China). The renal cryostat sections (7-mm) were prepared and perfused in 4% paraformaldehyde for 30 min. After permeabilized with 1% Triton X-100 for 5 min, incubated with 50 μl of TDT and Streptavidin-Fluorescein at 37 °C for 30 min, nuclei were performed with 4′, 6-diamidino-2-phenylindole (DAPI, Biyuntian biological technology, China).

### Flow cytometric analysis

Since apoptosis analysis cells were performed with Annexin-V-FITC Apoptosis Detection Kit (BestBio, China), samples from different groups were collected by trypsinization, and washed twice with cold phosphate buffered saline (PBS) buffer. Before analyses were performed on BD LSR flow cytometer (BD Biosciences), cells were resuspended in 400 μl Annexin-V binding buffer, added with 5 μl Annexin-V-FITC cultured at 2–8 °C for 15 min, and then added with 10 μl propidium iodide (PI) cultured at 2–8 °C for 5 min in dark.

### Small interfering RNA silencing

Cells were transfected with 100 nM of small interfering RNA (siRNA) using Lipofectamine 2000 (Invitrogen, CA, USA) according to the manufacturer’s instructions. The oligonucleotide sequences were as follows: BMP-7 siRNA (h), 5′-CGGAAGUUCCUGUAAUAAAdTdT-3′ for the sense strand and 5′-UUUAUU ACAGGAACUUCCGdGdG-3′ for the antisense strand; BMP-7 siRNA (m). A negative scrambled siRNA (General Biosystems, China) was used in parallel. Cells were cultured at 37 °C for 6 h, and then, Western blot was used 48 h after siRNA transfection.

### Statistical analysis

Data are represented as mean ± SD. Statistical analysis was performed using ANOVA followed by Student’s t-test. For changes in mRNA or protein levels, ratios of mRNA (relative expression) and protein (densitometric values) to respective house-keeping controls were compared. Significance was defined as p < 0.05.

## Additional Information

**How to cite this article**: Ma, T. *et al.* A potential adjuvant chemotherapeutics, 18β-glycyrrhetinic acid, inhibits renal tubular epithelial cells apoptosis via enhancing BMP-7 epigenetically through targeting HDAC2. *Sci. Rep.* **6**, 25396; doi: 10.1038/srep25396 (2016).

## Supplementary Material

Supplementary Information
