# Supplementary material for: A potential adjuvant chemotherapeutics, 18β-glycyrrhetinic acid, inhibits renal tubular epithelial cells apoptosis via enhancing BMP-7 epigenetically through targeting HDAC2
Source: Sci Rep. 2016 May 5;6:25396. doi: 10.1038/srep25396 (PMC4857087; doi:10.1038/srep25396)

Supplementary information

A potential adjuvant chemotherapeutics, 18β-glycyrrhetinic acid, inhibits renal tubular epithelial cells apoptosis via enhancing BMP-7 epigenetically through targeting HDAC2

Taotao Ma, Cheng Huang, Xiaoming Meng, Xiaofeng Li, Yilong Zhang, Shuai Ji, Jun Li*, Min Ye*, Hong Liang*

Table S1 Kinetic parameters for the binding of 18βGA to HDAC2

Ligand	Analyte	Ka($M^{-1}S^{-1}$)	Kd(S^{-1})	Kd(M)
HDAC2	18βGA	4.988×10^{2}	3.058×10^{-3}	6.131×10^{-7}

Table S2. Antiproliferative activity of glycyrrhizic acid (GA) and 18β-glycyrrhetinic acid (18βGA) against the mTEC and HK2 cell lines.

Comp.	IC_{50}/mM[a]	
	mTEC	HK2
GA	1.525±0.054	1.729±0.011
18βGA	0.089±3.194	0.073±0.520

[a] Each IC_{50} value is the mean ± SE from three experiments (n=3).

Supplementary Figure legends

Figure S1. The chemical structures of Glycyrrhizic acid (GA) and 18β-glycyrrhetinic acid (18βGA).

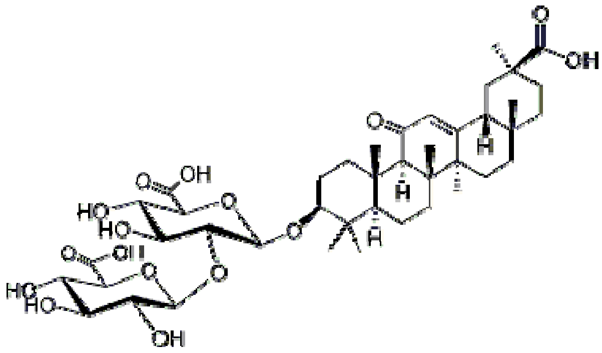

Glycyrrhizic acid (GA)

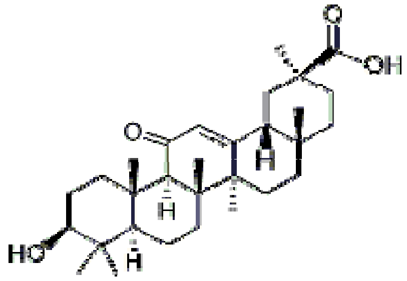

18β-glycyrrhetinic acid (18βGA)

Figure S2. Effect of TSA on expression of BMP-7 *in vitro*.

Western blot analysis of TSA and 18βGA on expressions of BMP-7 induced by CP in HK-2 cells and mTEC cells. Data are represented as mean ± SD of three independent experiments. *p < 0.05, **p < 0.01 vs. control group, #p < 0.05, ##p < 0.01 vs. CP alone.

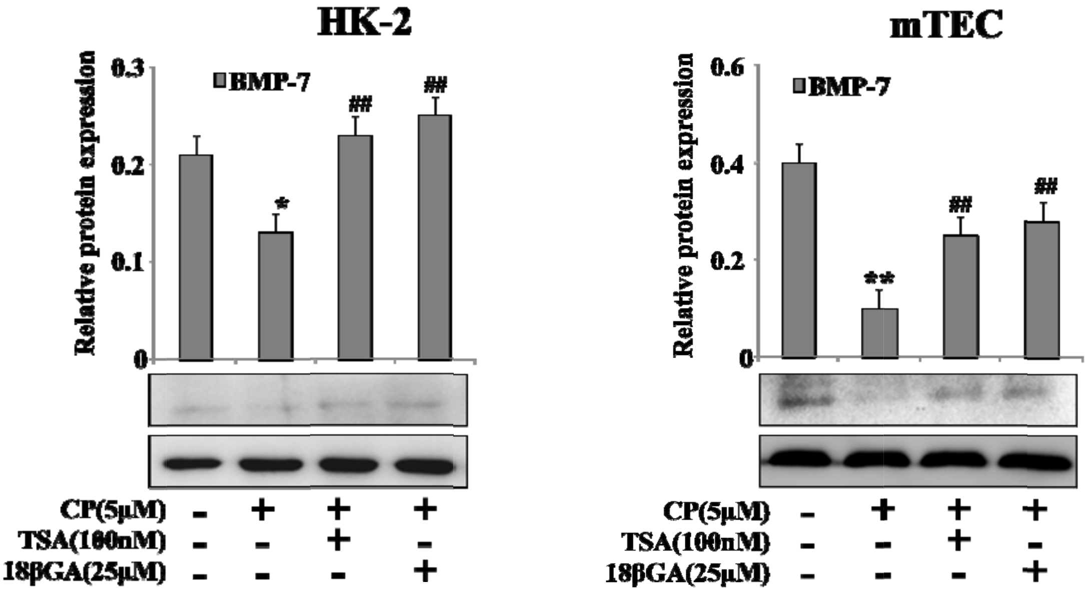

Figure S3. A proposed kidney protective signaling pathway of GA.

18βGA inhibits apoptosis of renal tubular epithelial cells via enhancing level of BMP-7 epigenetically through targeting HDAC2,

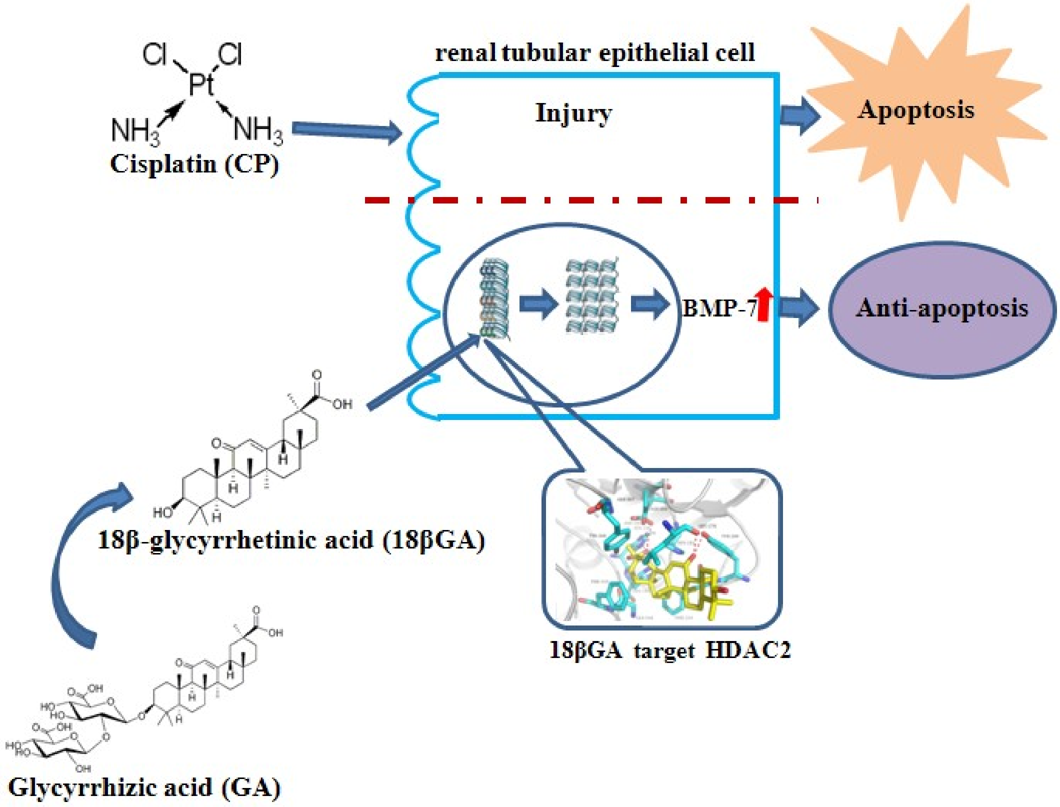

Supplement: Supplementary Information [file srep25396-s1.pdf]
